# “Kind of Feel Like the Lone Outpost”: A Qualitative Exploration of Barriers to Professional Development for Rural Primary Care Providers at the Veterans Health Administration

**DOI:** 10.1111/jrh.70206

**Published:** 2026-08-18

**Authors:** Karla L. Miller, Jennifer Hale‐Gallardo, Jaime R. Wilson, Melissa L. Swee, L. Ida Tovar, Bret Hicken

**Affiliations:** ^1^ Salt Lake City VA Healthcare System Salt Lake City Utah US; ^2^ Division of Rheumatology University of Utah Department of Medicine Salt Lake City Utah US; ^3^ VHA Office of Rural Health Veterans Rural Health Resource Center Salt Lake City Utah US; ^4^ VHA Office of Rural Health Veterans Rural Health Resource Center Iowa City Iowa US; ^5^ Center for Access & Delivery Research and Evaluation (CADRE) Iowa City VA Health Care System Iowa City Iowa US; ^6^ University of Iowa College of Nursing Iowa City Iowa US; ^7^ Division of Nephrology Department of Medicine University of Iowa Iowa City Iowa US; ^8^ Division of Geriatrics Department of Medicine University of Utah Salt Lake City Utah US

**Keywords:** needs assessment, primary care, professional development, rural workforce, Veterans Affairs

## Abstract

**Introduction:**

Ongoing professional development (PD) is vital to high‐quality healthcare, workplace satisfaction, and the retention of rural primary care providers (PCPs). However, the barriers to PD experienced by rural PCPs remain underexplored. This evaluation examined barriers to PD engagement among rural Veterans Health Administration (VHA) PCPs.

**Methods:**

A descriptive, cross‐sectional qualitative design was employed to explore barriers to PD participation. Participants included nine rural VHA PCPs, six ambulatory care leaders, and three facility leaders. Recruitment continued until data saturation was reached. Rurality was determined using Rural‐Urban Commuting Area (RUCA) codes. Semi‐structured interviews and focus groups were conducted, recorded, transcribed, and analyzed using a rapid qualitative framework approach.

**Results:**

Participants represented diverse rural regions and disciplines. PCPs included physicians, a nurse practitioner, a physician assistant, and a pharmacist. Six themes emerged as barriers to PD: (1) ineffective or delayed communication of PD opportunities; (2) rural clinic capacity constraints, including understaffing with inadequate patient coverage and insufficient protected time; (3) high opportunity costs; (4) limited geographic access; (5) professional isolation; and (6) perceived devaluation of the PCP role.

**Conclusions:**

This qualitative evaluation provides new insight into how rurality and its associated barriers influence access to and engagement in professional development among rural PCPs. Findings highlight the need to improve communication, ensure protected time, and expand access to PD while mitigating professional isolation. Understanding these barriers is crucial for designing feasible interventions that enhance PD engagement, strengthen the rural workforce, and improve quality of care for rural Veterans.

## 1 Introduction

Professional development (PD) for healthcare providers aims to enhance competencies in leadership, collaboration, communication, ethics, and technical expertise [1]. PD is critical for integrating evidence‐based practices, building learning health systems, and fostering high reliability organizations [2]. Provider engagement in PD also contributes to workplace satisfaction, improves retention and reduces burnout [3, 4, 5, 6, 7, 8, 9]. This is especially key for the recruitment and retention of primary care providers to rural areas [8, 9, 10]. One‐third of the 9.1 million US Veterans enrolled in the VA live in rural areas, and a majority (about 66%) rely on the Veterans Health Administration (VHA) for their health care, compared with 47% of their urban counterparts [11].

Rural Veterans are generally older, have more chronic conditions, and depend heavily on the network of more than 800 VHA Community‐Based Outpatient Clinics (CBOCs) [11, 12]. Persistent shortages of rural PCPs negatively affect primary care provision for both the private sector and the VHA [9, 13, 14, 15, 16], with rural VHA CBOCs experiencing higher provider turnover, and burnout [15, 17], resulting in many incomplete rural Patient‐Aligned Care Teams (PACTs) [16]. A recent national analysis of VHA PCPs found that separation from employment was more likely at systems with high burnout [18], negatively impacting workforce stability [17].

Burnout is especially high among rural VHA PCPs at non‐teaching sites, according to a national study [17]. Rural physicians more broadly have been described as carrying broader scopes of practice and greater responsibility for complex patient needs with limited support [19, 20]. Workplace‐climate factors, including workload, team staffing, and perceived organizational support, are among the most influential modifiable predictors of burnout among VHA PCPs [21], particularly for those who coordinate a high volume of Community Care referrals outside the VHA [22]. Together, these factors are consistent with the broader literature linking understaffing, high patient demand, and administrative burden to clinician burnout [21, 23]. Within VHA primary care specifically, burnout has also been associated with organizational and care‐delivery challenges [24, 25], which has direct consequences for Veteran access to primary care in rural communities [26]. Primary care access is associated with better management of chronic disease and lower mortality [27]. Turnover and persistent understaffing in rural VHA clinics can disrupt continuity of care, increase provider burden, and require Veterans to travel longer distances to obtain healthcare [13, 15, 22, 28]. Retention of experienced rural PCPs is therefore central not only to workforce stability but also to equitable access to and quality of care for rural Veterans.

Professional development is associated with increased workplace satisfaction, reduced burnout, and improved retention among rural providers [6, 10, 26, 29]. International guidance identifies ongoing PD as integral to rural and remote health workforce retention strategies [30]. The VHA offers several national and facility‐level PD initiatives [31], yet access remains uneven for rural providers due to differences in site complexity, local resources, academic affiliation, and leadership priorities. Additionally, program sizes and reach vary across the VHA, and PD effectiveness as a stand‐alone intervention is difficult to measure in light of its interconnectedness with other retention factors. Rural clinicians consistently report greater difficulty accessing PD than their urban counterparts [10, 32], and have been found to participate less in formal continuing medical education than urban peers, largely because of structural barriers such as distance, cost, time, limited local access, and professional isolation [33, 34], with more recent work continuing to support these findings [35]. Though these barriers are well documented, understanding how rural VHA PCPs and their leaders perceive barriers to PD is essential for designing interventions that support this important workforce. This qualitative evaluation explores barriers to PD among rural VHA PCPs.

## 2 Methods

### 2.1 Design, Evaluation, Setting, and Participants

This evaluation employed a descriptive, cross‐sectional qualitative design [36] as phase one of a sequential, 3‐year quality improvement (QI) project to explore barriers and facilitators to professional development (PD) among VHA primary care providers (PCPs). Subsequent phases, including a national survey and an intervention, will be reported separately.

The overarching question for this qualitative phase was: “What are the key barriers to professional development engagement among rural VHA PCPs and their leaders?”

Eligible participants were VHA PCPs employed at rural CBOCs, and ambulatory care or facility leaders with at least 6 months of experience; those practicing exclusively in urban sites or with shorter tenure were excluded. Rurality was defined by rural‐urban commuting area (RUCA) codes. A purposive sample was recruited through email invitations [37] and limited snowball sampling to maximize diversity of disciplines and geographic regions, until data saturation was achieved (no new code themes in three consecutive interviews). All recruitment was handled by team members with no supervisory relationship with participants and included assurances of voluntariness and confidentiality.

All invited individuals participated, resulting in a final sample of 18 participants: nine rural PCPs, six ambulatory care leaders, and three facility leaders, together representing frontline, managerial, and organizational levels. PCP participant demographics, including discipline, years of service, and region, are shown in Table 1.

**TABLE 1:** Demographic characteristics and professional development interests of rural VA primary care provider participants (*n* = 9).

Gender	Female	Female	Male	Female	Female	Male	Female	Female	Female
Degree type	MD	MD	MD	MD	NP	MD	PharmD	MD	PA
Years in VA	23	13	4	17	3	11	21	<1	<1
Years in rural CBOC	23	13	4	11	3	11	21	<1	<1
Years since training complete	23	No resp	14	21	9.5	11	24	<1	15
Rural or small town upbringing	Yes	Yes	Yes	Yes	No	Yes	Yes	Yes	Yes
Geographic Region	Midwest	South	West	South	West	South	South	Northeast	Midwest
PD interests	CS, QI	L, QI	QI, L	CS, L, QI	CS, QI	L	E, QI	E, L, QI	CS
PD important for recruitment	No	No	No	No	No	No	No	Yes	Yes
PD important for retention	Yes	Yes	Yes	Yes	Yes	No	Yes	Yes	Yes
Gender, *n* (%)			Female: 7 (78); male: 2 (22)						
Clinical discipline, *n*			Physician (MD): 6; nurse practitioner: 1; physician assistant: 1; PACT clinical pharmacist: 1						
Years at current rural VA CBOC, median (range)			11 (<1–23)						
Years since completion of clinical training, median (range)^a^			14.5 (<1–24)						
Rural or small‐town upbringing, *n* (%)			8 (89)						
Geographic regions represented			Midwest, Northeast, Southeast, and Mountain West						
Primary PD interests reported, *n* ^b^			Quality improvement: 7; leadership: 5; clinical skills: 4; education or preceptorship: 2						
PD important for retention, *n* (%)			8 (89)						
PD important for recruitment, *n* (%)			2 (22)						

*Note*. The pre‐interview demographic questionnaire was administered only to the nine rural PCP participants. Ambulatory care and facility leader participants did not complete the demographic questionnaire; their individual characteristics are not reported here to protect anonymity given the small size of each leader group.

Abbreviation: CS = clinical skills, QI = quality improvement, L = leadership, E = education or preceptorship; PD = professional development; CBOC = Community‐Based Outpatient Clinic; PACT = Patient‐Aligned Care Team.

^a^ One participant did not report years since training (*n* = 8).

^b^ Participants could report more than one area of interest; counts therefore sum to more than *n* = 9.

### 2.2 Reflexivity

The initial team included two co‐authors, contributing complementary clinical, operational, and methodological expertise. The lead author is a physician directing the VHA Rural Scholars program with deep familiarity with the rural CBOC context under evaluation; the co‐lead author contributed expertise in qualitative methods and rural health services research. Both co‐lead authors were actively involved in all stages, including study design, data collection, analysis, and interpretation; reflective discussions were held throughout data collection and analysis, with analytic decisions documented to enhance rigor and minimize bias. Additional team members contributed substantively through critical review, content development, and manuscript revision. The protocol and reporting adhered to SRQR standards (Supplement A) [38].

### 2.3 Data Collection

Semi‐structured interview guides were informed by literature review and operational partner feedback, and pilot tested with a small group of VHA providers. The final guides included open‐ended questions to explore provider engagement with, barriers to, and overall perspectives about PD (Supplements C–E). Data were collected virtually via Microsoft Teams (September 2021 to July 2022). Twelve individual interviews (nine PCPs, three facility leaders) and one focus group (six ambulatory care supervisors) were conducted by trained team members; interview length averaged 45 min and focus groups length was approximately 60 min. PCP interviews addressed potentially sensitive topics individually to maintain confidentiality, while focus groups facilitated shared managerial perspectives. Facility leaders were interviewed separately to preserve candor among the ambulatory care leaders. Demographic questionnaires were completed by PCPs prior to interviews (Supplement B). All sessions were recorded. No incentives were offered.

### 2.4 Data Analysis

Audio recordings were transcribed verbatim by a professional transcription service and checked for accuracy. Data analysis employed a rapid qualitative framework method [39, 40]. First, an excel‐based matrix was developed, with columns representing key domains from the interview guide (provider engagement, barriers, facilitators) and rows for each participant or focus group. Each transcript was reviewed, and relevant content was summarized directly into the corresponding matrix cell according to domains (deductive coding). As analysis progressed, the matrix was iteratively refined to incorporate new themes that emerged inductively from the data but were not captured by the original guide. This process supported both the systematic organization of anticipated themes and flexibility to capture novel insights. Initial coding and matrix population were performed by one author, with a second author reviewing for consistency and resolving discrepancies. Regular team meetings were held to reconcile differences, discuss emerging findings, and ensure multiple perspectives informed the analysis. Member checking consisted of presenting preliminary themes to clinical, educational, and workforce development teams, including rural primary care providers.

## 3 Results

### 3.1 Sample Characteristics

A total of eighteen participants were interviewed, including nine rural PCPs, six ambulatory care leaders, and three facility leaders. The range of years since the completion of rural PCP medical training varied from less than 1 year to 24 years; the span of years in practice at a rural VHA clinic for this cohort ranged from less than 1 year to 23 years. Only one participant completed a rural health pathway as a rural workforce incentive, during medical training. Two PCPs had prior or ongoing military experience, and the majority reported a rural or small‐town upbringing. All but one PCP indicated that PD was important for their retention. Notably, only the two PCPs who had been in their rural VHA positions for less than 1 year indicated PD was important to their recruitment (see Table 1 for detailed characteristics of PCPs).

Data analysis yielded six primary themes that highlight key barriers hindering rural PCP participation in PD: (1) ineffective or delayed communication of PD to rural PCPs; (2) rural clinic capacity constraints, with subthemes of understaffing, inadequate patient coverage, and insufficient protected time for PD; (3) higher opportunity costs for rural PCP participation in PD; (4) limited geographical access to PD; (5) professional isolation for rural providers; and (6) Perceived devaluation of the PCP role.

#### 3.1.1 Ineffective or Delayed Communication of PD to Rural PCPs

Many participants reported delayed and ineffective dissemination of PD opportunities, resulting in missed opportunities to engage and a lack of awareness of where to obtain information on PD. One PCP explained: “I don't even know where I would get information” [Female MD, PCP, Northeast]. Another found it odd that she could learn more about opportunities through external conferences rather than internal PD communications: “I don't know who to talk to, where you find those opportunities. I learn more about what is going on in Whole Health or Integrative Medicine at conferences outside the VHA. That's just weird to me” [Female MD, PCP, Midwest].

Facility leaders expressed uncertainty about how to locate career growth opportunities for providers: “Actually no, if you ask me where I would go to find out about these things, I couldn't tell you. Most of it I think is happenstance” [Male MD, Facility Leader].

A lack of timely communication of PD opportunities also impeded participation due to fully booked clinics. One participant expressed frustration that something as brief as a webinar was communicated with inadequate notice: “I would feel completely frustrated when I would receive communication about a webinar that was happening in a week, and my schedule is already completely booked” [Female MD, PCP, Midwest]. Ambulatory care leadership echoed these concerns and recommended timeliness in sharing offerings:

“Show consideration–if you are sending the opportunity the week before, that may be okay for someone with a lot of administrative time, but if I'm in clinic I have to give 45 days’ advanced notice. It seems inconsiderate, it comes off that way” [Male MD, Ambulatory Care Supervisor].

#### 3.1.2 Rural Clinic Capacity Constraints

##### 3.1.2.1 Understaffing and Inadequate Patient Coverage for Rural Clinics

Understaffing was a major challenge to rural PCPs’ ability to pursue PD. One PCP explained, “Unfortunately for CBOCs, they are always short‐staffed. We do have a float provider here, but she's hardly ever here because she's floating around at other places” [Female PA, PCP, Midwest].

Understaffing of healthcare teams had a direct impact on patient coverage in a smaller clinic, where the absence of one provider could present significant stress. One PCP stated, “If you are in a small clinic, you don't want people resenting you [for doing PD]” [Female MD, PCP, Midwest]. Another emphasized this by saying: “[I need] more coverage of my [patient] panel to do any sort of training [PD]” [Female MD, PCP, Northeast].

##### 3.1.2.2 Insufficient time for PD for Rural PCPs

Rural workload demands decreased the capacity for PD engagement. A nurse practitioner expressed she would need “…time to implement project or teach or whatever because in a clinic day there isn't ‘spare’ time” [Female NP, PCP, West]. Echoing this, an ambulatory care supervisor explained, “If it requires significant time–more than an hour or two–they're trying to balance what they will gain from the training with their clinical load” [Female MD, Ambulatory Care Supervisor].

#### 3.1.3 Higher Opportunity Costs for Rural PCP Participation in PD

Participating in PD as a rural PCP involved opportunity costs, or trade‐offs. For one facility leader, the issue of dedicated time was: “… just a snippet in a much larger issue of how we balance several things patient panel size, patient demand, labor mapping, clinic grids, meeting the access measures, so that a provider's labor mapping is really valued” [Male MD, Facility Leader].

##### 3.1.3.1 High Rural Care Coordination Workload

Rural Veterans often seek diagnostic exams and attend consultations in their local communities rather than traveling to a VHA, increasing the demands of care coordination, and therefore, PCP workload. As a result, managing referrals and tracking external records increases the perceived opportunity costs for rural PCPs considering PD:

“It is very, very challenging to do well, to be a good [rural] primary provider. Care coordination is really challenging…it's really hard to read records from [non‐VHA] institutions that you're not part of and try to figure out what's going on…that type of care coordination is really challenging” [Female MD, PCP, Northeast].

Ambulatory Care leaders also related the challenges of the extra workload for their rural PCPs due to the reliance on specialists in the community:

“The other thing we have really, really come to appreciate is, the farther away your clinic is from your main VHA, the way higher likelihood that all of your specialists will be through CITC (community care). It's a huge workload that is not being studied that we know of, it's not being taken into account when it comes to [patient] panel size, and it just sucks up so much time and I think that it really needs to be looked at too, to keep it fair” [Female MD, Ambulatory Care Supervisor].

##### 3.1.3.2 Conflicting Metrics

Even in the case of fully staffed clinics, PCPs emphasized a conflict between seeing patients and applying new skills within rural clinics if the panel size wasn't adjusted. One participant described learning new clinical skills in a VHA CBOC as “tricky” because “the more you do, the more you do” [Male MD, PCP, West].

A second PCP summarized the issue at stake with conflicting metrics, saying:

“I was retrained on joint injections, knee injections, shoulder injections, and battlefield acupuncture, which was great, but I got so busy doing those things, that my access [metric] became poor. Patients absolutely would rather come to this clinic than travel to an orthopedic, so it's a great service, but there's a payoff” [Female MD, PCP, Midwest].

Facility leaders also acknowledged important tradeoffs facing rural PCPs. One facility leader reasoned that: “What you want is a provider to want to do these (PD) without being punished for taking the time” [Male MD, Facility Leader]. Others said it came down to a question of priorities and value. One participant noted, “If you are going to value it [PD], you have to demonstrate that value by offering the time [to do PD] and not being as focused on the numbers [patient quotas]” [Male MD, Ambulatory Care Supervisor].

##### 3.1.3.3 Burnout

Ambulatory Care leaders considered burnout a particular concern for rural PCPs and perceived that this was impeding PD engagement: “It's really hard when you have a fully paneled provider who is somewhat burnt‐out just seeing patients, it's really hard for them to be able to take advantage of the opportunities that the VHA does have to offer” [Female MD, Ambulatory Care Supervisor].

One PCP noted: “I'm not in a position where I think I could do an additional ‘something’” [Female MD, PCP, Northeast].

The high potential for burnout due to overburdened schedules impacted not only the feasibility of PD for rural providers but prevented the kind of thinking and planning that could impact long‐term career opportunities: “When you're crunched for time and have all of these other things going on, it's hard to think in the long term about career development” [Male MD, PCP, West].

#### 3.1.4 Limited Geographical Access to PD for Rural PCPs

A few participants reported that since COVID‐19, virtual PD is more accessible, but also raised challenges related to internet quality and being interrupted by team members when trying to engage in virtual synchronous and asynchronous PD. However, ambulatory care and facility leaders recognized that geographic barriers still impacted rural PCPs when in‐person attendance was required. One leader acknowledged the need for geographically accessible opportunities, emphasizing: “If it has to be in person, which is really great, having more diverse geographic opportunities would be helpful” [Male MD, Ambulatory Care Leader].

#### 3.1.5 Professional Isolation

An important barrier to PD was that of professional isolation. Key subthemes included limited professional networks; disconnect between rural clinics and main campus; limited developmental relationships; unclear pathways to career advancement; and devaluation of the PCP role.

##### 3.1.5.1 Subtheme: Lack of Professional Networks

All participants reported the absence of structured opportunities to connect with peers. One PCP said he “felt ‘surprised’ of the non‐existence of VHA CBOC conferences that would allow him to learn about what his colleagues were doing…even just on a regional level (in order to) increase collegiality” [Male MD, PCP, West].

Another physician explained that she didn't “know how to participate with other rural CBOCs” [Female MD, PCP, Northeast]; a nurse practitioner underscored the professional isolation, explaining, “You feel pretty isolated [working in a rural CBOC] (so) opportunities to network would be something I am looking for” [Female NP, PCP, West].

This isolation was acknowledged by leaders, with one ambulatory care supervisor reflecting that “[rural PCPs] kind of feel like the lone outpost” [Male MD, Ambulatory Care Supervisor].

##### 3.1.5.2 Subtheme: Disconnect Between Rural Clinics and the Main Campus

The disconnect between rural clinics and VHA main campuses or academic affiliates surfaced among several participants. One PCP emphasized that this did not apply to all the VHA; however, for rural clinics there was a sense of a “disconnect between the ‘boots on the ground’ people, and those doing research, QI, [and] administration.” This participant underscored that “intentionality is needed to connect people in power, or research, to those people working in rural clinics” [Female MD, PCP, Northeast].

An ambulatory care supervisor also pointed to a gap in access to PD based on rurality: “We have an academic affiliate, and providers there have much more protected time, do less clinic, and have better opportunities for professional advancement” [Male MD, Ambulatory Care Supervisor]. This leader was involved in initiatives to overcome these barriers for rural providers by expanding CME offerings but also acknowledged that “it's a hard barrier to overcome.”

##### 3.1.5.3 Subtheme: Limited Developmental Relationships

In a context where PCPs grapple with professional isolation, the concept of developmental relationships took on added significance. Professional mentorship was perceived to be an elusive asset. One PCP said: “Mentorship is always good, [but] I don't know anybody who really gets mentored as a PCP” [Female MD, PCP, Northeast].

A recurring theme was the role of leadership in creating an environment that encouraged healthcare professionals to engage in PD and grow their careers. A sense of longing for individualized career guidance was emphasized by one PCP: “There's no mentorship, [but] that would be awesome if someone sat with you and went over your career goals.” [Female MD, PCP, Midwest]. Another desired more support for QI projects, for example, if a leader would say: “…’X provider is doing their project and we're having a special meeting to talk about it” [Female NP, PCP, West]. Another PCP mentioned the importance of leaders who can pick out the talent and say, “I know you're busy, but I'd like to see you think about” [this PD opportunity] [Male MD, PCP, South].

##### 3.1.5.4 Limited Pathways for Rural Career Advancement

All PCPs except for one indicated that PD was important for their retention as a rural provider. Despite this emphasis on PD for retention, a prevailing sentiment among PCPs was the difficulty in advancing careers in rural settings. As one PCP stated, “Advancement is hard because it typically requires a move, other than designation as a medical director [of CBOC]” [Male MD, PCP, West]. Participants understood career progression to necessitate relocation to urban areas or to larger healthcare facilities. Many faced a career advancement conundrum of “no way to move up that way [career advancement] unless you go into management” [Male MD, PCP, South].

Limited existing career paths for rural PCPs prompted some PCPs to seek opportunities at the national level, believing this would garner recognition and potentially lead to career advancement. One physician shared this perspective, stating, “The program I am in is national and I thought that if I engaged in a national program, it would be harder for local leadership to ignore that. It would be harder for them to say, ‘no you stay where you're at’” [Male MD, PCP, South].

A rural facility leader elucidated that leadership pathways that focus on clinical team leadership or clinical initiative leadership are missing compared to management leadership positions that often require moving to a more urban area; this negatively impacts rural providers who do not have an opportunity to advance locally within their roles, which he perceived as a barrier to PD.

#### 3.1.6 Perceived Devaluation of PCP Role

PCPs relayed concerns about a larger culture that treated their work as a bureaucratic duty, lowering motivation for PD. One rural PCP remarked,

“I understand that this is a bureaucratic system and that there is a hierarchy, and it is important to follow the rules. But I also think there is definitely room for improvement to recruit people to more than ‘just a job’” [Male MD, PCP, South].

Other providers felt depersonalized in their roles expressing, “As much as I love my job, I feel like a factory worker, and my other docs have said that” [Female MD, PCP, South]. Another added, “I've just been a ‘worker bee’ in primary care for the last twenty‐some years” [Female MD, PCP, Midwest].

Leaders also acknowledged the importance of PD in professional satisfaction for rural PCPs, and the cultivation of fulfilling work. One ambulatory care supervisor stated, “Some of our providers start to feel like there's some depersonalization, and it's really just somebody doing the job and someone counting the beans” [Female MD, Ambulatory Care Supervisor]. Leaders underscored how professional fulfillment was key to PCPs, one emphasizing that “people want professional satisfaction more than they want money” [Male MD, Facility Leader].

## 4 Discussion

This qualitative evaluation of barriers to professional development addresses a profoundly important yet understudied issue: the barriers faced by rural VHA PCPs in pursuing and engaging in professional development, and suggests that many complex, intersecting factors impact how rural providers perceive barriers to engaging in PD. Some of these factors are unique to the VHA, while others have also been substantiated in rural workforce studies outside the VHA.

Rural clinic capacity constraints were a major theme in our study, with subthemes of understaffing with inadequate panel coverage, and insufficient time impacting capacity of rural providers for professional development. In our study, rural VHA PCPs perceived their PACTs to be frequently understaffed, which caused them to be concerned about the feasibility of taking time off for PD. Challenges with staffing rural PACTs in the VHA have been substantiated in the literature [16], though the impact on the pursuit of PD is not previously described. Conversely, understaffed VHA primary care teams and turnover are associated with inadequate resources that impede effective team‐based care [16] and can contribute to burnout [21]. Insufficient time for the PD itself or to implement new skills was identified as a barrier to PD and is substantiated as a barrier for both urban and rural PCPs [41, 42], though rural providers appear to be more impacted by this barrier [42].

Compounding these challenges, our participants emphasized the high opportunity costs tied to serving as the frontline of community care coordination for rural Veterans, conflicting performance metrics, and feelings of burnout impacting the motivation to pursue PD. Previous studies have confirmed that rural VHA providers incur a higher care coordination burden [22] and other studies indicate lower engagement in PD related to this burden [43], as well as increased burnout [22]. Some participants in our study successfully pursued PD in specialty services, such as joint injections, to reduce travel burden for rural Veterans while providing access to a much‐needed specialty procedure. However, providing this specialty service resulted in fewer available slots for the PCPs’ assigned primary care patients, causing their Veteran access metrics to decrease and disincentivizing them from pursuing additional PD for these specific specialty skills. Misaligned metrics and mandates have been found to undermine provider motivation, autonomy, and satisfaction [44, 45, 46], which contribute to burnout, but they may also inadvertently impact the motivation to pursue necessary training to provide innovative services at rural VHA clinics that benefit rural Veterans.

Geographic barriers and professional isolation have been commonly reported and associated with lower recruitment and retention in rural areas [9, 47, 48]. In our study, rural VHA PCPs and leaders identified how professional isolation impacted their professional development experience via limited access to professional networks, main VHA campuses, mentorships, and pathways for career advancement. Healthcare professional mentorship has been associated with research, teaching, clinical skill development, networking, and career advancement [49] but is a challenging resource to provide in rural areas [50]. Though increasing virtual synchronous and asynchronous PD options were appreciated, they represented only a partial solution, as barriers such as uninterrupted protected time, panel coverage, and high opportunity costs still applied. Moreover, other barriers such as the timely communication of PD opportunities, were perceived by participants to disproportionately affect rural PCPs. Timely and effective communication is essential in the complex healthcare environment impacting quality and cost of healthcare [51, 52], and improved marketing and publicity of PD was recommended by healthcare providers in a recent national survey [32].

A central insight from our findings is that rural PCPs experience a combination of barriers that go beyond logistical obstacles. Professional isolation plays an outsized role, impacting rural PCP PD experience via limited access to professional networks, main VHA campuses, mentorships, and pathways for career advancement, but also affecting the sense of professional fulfillment for, and the retention of, providers in rural areas [9, 24, 29]. Participants and leaders reported a perception that career advancement generally requires moving to a more urban setting and engaging in managerial activities rather than recognizing or rewarding clinical leadership or innovation within rural practice. Notably, there was a clear sense that opportunities for growth and advancement were more available at main VHA campuses than at outlying rural clinics: a perception that further dampened enthusiasm for pursuing PD. Studies of medical students and graduates have identified a perception of limited opportunity for PD and career advancement as a factor negatively influencing their intention to practice rurally [10, 29].

PCPs consistently expressed feeling undervalued, likening themselves more to “worker bees” or “factory workers” than independent professionals, which impacted their motivation to seek further professional growth. Other studies have also reported perceived undervaluation of the primary care profession and an imbalance between increasing responsibilities and decreasing autonomy among PCPs, where the growth of organizational directives, metrics, and mandates begins to eclipse provider autonomy [23]. Other hierarchical organizational cultures associated with public sector work have been found to negatively impact PCP professional satisfaction, and even small changes toward a healthcare system that is more aware of and responsive to the needs of rural PCPs may promote improvement in PCP satisfaction, which is associated with retention [53, 54].

Altogether, our evaluation highlights how the interplay of geographic isolation, system‐level organizational pressures, care coordination burdens, and professional isolation create a uniquely difficult environment for rural VHA PCPs pursuing PD. These findings matter deeply as VHA already faces challenges in attracting and retaining rural PCPs: professional isolation and barriers to PD are closely linked to burnout and intent‐to‐leave, which are well‐documented drivers of both provider turnover and lower patient care quality [55, 56, 57]. Furthermore, burnout combined with professional isolation may create a reinforcing cycle where isolation increases burnout [58], burnout reduces career engagement [55], and reduced engagement further exacerbates isolation, negatively impacting care for a particularly vulnerable Veteran population. Rural VHA PCPs manage Veterans’ complex medical and psychosocial needs, and this skill cannot be easily backfilled, further increasing the importance of supporting and developing this unique workforce.

Finally, while our findings align with prior research outside the VHA on professional isolation and access to PD [9, 10, 33, 34], our focus on rural VHA PCPs highlights unique intersections between rurality, system‐level metrics, and care coordination within the VHA system [15, 22, 44], as well as the complex relationship with provider burnout [17, 21]. These findings bring to light the need for targeted and accessible PD opportunities for rural VHA providers, ones that recognize and address their realities, reward local leadership and innovation, and provide pathways for career progression that do not necessitate leaving rural practice or assuming solely administrative roles. Research shows that promoting more effective communication of, access to, and protected time for relevant PD, along with peer networking, and mentorship, supports PCP engagement and helps buffer against burnout [21, 52].

One limitation of this evaluation is its reliance on a small sample of providers and leaders, which affects the generalizability of the findings to the broader population. Nonetheless, a sampling effort was made to include participants from various regions throughout the country. We took steps to strengthen rigor and trustworthiness, including peer debriefing and member checking to minimize interpretative bias. Nevertheless, complete elimination of subjectivity is not possible. While interview guides were carefully crafted to minimize leading language, there is still the possibility that phrasing may have inadvertently influenced participant responses. Additionally, despite interviewer training, subtle interviewer effects cannot be entirely ruled out. Many of these limitations were subsequently addressed through a follow‐up survey, which utilized psychometric methods and included a larger, more representative sample of providers nationwide to build upon and extend the qualitative findings.

## 5 Conclusion

This qualitative evaluation within the largest healthcare system in the US highlights how rural VHA PCPs face overlapping barriers to PD, including geographic isolation, limited mentorship, substantial administrative burdens, and lack of opportunities for advancement in rural clinical leadership roles. Many of these challenges intersect with and are exacerbated by professional isolation and burnout, which are interrelated drivers of workforce instability in rural healthcare settings, threatening rural provider retention. Future efforts should examine how to integrate rural VHA PCPs into local and system‐wide professional networks that can strengthen rural provider opportunities for and engagement in PD, promote workforce stability, and ultimately, the quality of care rural Veterans receive.

## Funding

This work was supported by the VHA Office of Rural Health.

## Conflicts of Interest

The authors declare no conflicts of interest.

## Disclaimer

The views expressed in this article are those of the authors and do not necessarily reflect the position or policy of the Department of Veterans Affairs or the US government.

## Disclosure

During the preparation of this work for resubmission, the authors acknowledge the use of Claude AI and VA GPT for formatting and copy‐editing. No AI‐generated scientific content, results, analysis, interpretation, or conclusion was incorporated into this work, and the authors take full responsibility for the content.

## Ethics Statement

This evaluation was conducted as a quality improvement (QI) needs assessment under the VA and University Institutional Review Board's operational guidelines and was determined to be “non‐research” by the VHA. Participation was voluntary, verbal consent was obtained from all participants, and identifiers were promptly removed from all transcripts. Data were stored on secure VHA servers in accordance with institutional data protection policies. All data were de‐identified and participants were assured that quotes used in reports would not identify individual respondents.

## Supporting information




**Supporting File 1**: jrh70206‐sup‐0001‐SuppMat.docx.

**Supporting File 2**: jrh70206‐sup‐0002‐SuppMat.docx.

**Supporting File 3**: jrh70206‐sup‐0003‐SuppMat.docx
