# Supplementary material for: “Kind of Feel Like the Lone Outpost”: A Qualitative Exploration of Barriers to Professional Development for Rural Primary Care Providers at the Veterans Health Administration
Source: J Rural Health. 2026 Aug 18;42(3):e70206. doi: 10.1111/jrh.70206 (PMC13483286; doi:10.1111/jrh.70206)
Supplement: Supplementary file 1 [file JRH-42-0-s001.docx]

Supplement B: Pre-Interview Survey Questionnaire

Introduction

Your answers to the following questions will help us prepare for this conversation.  Thank you in advance for your responses to these; we are looking forward to our phone call.

Please write NA for any questions that are not applicable.

Professional Background

1. What are your professional credentials?
2. How many years have you been working as a health care provider in the VA?
3. How many years have you been working in a rural VA community-based outpatient clinic (CBOC)?
4. How many years since you finished your highest level of training?
5. For physicians, what residency training did you complete (I.e. Family Medicine, Internal Medicine)
6. For advanced practice providers, what post-graduate training program did you complete (i.e. Family Practice, Internal Medicine, Women’s Health, Behavioral Health)?
7. Did your health professions training offer a structured rural health pathway?

Military Service Background

8. Have you ever served in the military?
9. Did your military service financially support your health professions education?

Personal Background

10. Did you grow up in a rural area?

Professional Development Background

11. What professional development opportunities at VA are you aware of?
12. Which VA professional development opportunities, if any, have you pursued?
13. On a scale of 1-5, overall, rate your satisfaction with VA professional opportunities you have participated in (with 1 being the lowest level of satisfaction and 5 being the highest level of satisfaction).
14. What, if any, professional development opportunities outside of the VA have you pursued?
15. On a scale of 1-5, overall, rate your satisfaction with the professional opportunities you have participated in outside the VA (with 1 being the lowest level of satisfaction and 5 being the highest level of satisfaction).
16. What Quality Improvement (QI) initiatives at the VA have you participated in? (for each, indicate whether served as lead or team member)
17. On a scale of 1-5, overall, rate your satisfaction with QI initiatives at the VA (with 1 being the lowest level of satisfaction and 5 being the highest level of satisfaction).
18. On a scale of 1-5, rate how relevant at you feel quality improvement initiatives are to your current clinical practice in the VA (with 1 being the lowest level of relevance and 5 being the highest level of relevance).
19. How would you rate the communication of professional development opportunities available to you as a rural VA provider?

Supplement C: INTERVIEW GUIDE FOR RURAL VA CBOC PROVIDERS

Introduction

Welcome, purpose of the study, confidentiality statement, verbal consent.

Main Questions

1. Please tell me what drew you to practice in a rural community? (practice-focused)

a. Probe: What do you enjoy about serving rural Veterans?
b. Probe:  What keeps you working at a rural VA CBOC?

2. What types of professional development opportunities interest you most?
   a. [If responded on survey that pursued any professional development opportunities in the VA]: What made these professional development opportunities appealing to you?

3. What helped make it possible for you to pursue the professional development opportunity through the VA?  (If pursued outside the VA) What made these opportunities appealing to you?
4. During your time working in the VA, what needs related to your own professional or career development have come up for you?
   a. Probe: Are there any skills you would like to develop further?  [If yes,] what are these?

Probe: [If no], are there any experiences that you feel would help you advance in your career?

b. How did the VA’s professional development prospects impact your decision to

take the position in the first place?

5. How does availability and access to professional development opportunities impact your decision to continue in your VA position?
   a. What would make it more likely for you to pursue professional development

opportunity offered through the VA?

6. What barriers have you experienced in taking advantage of professional development opportunities?
   a. Probe: Financial barriers (personal or institutional)?
   b. Probe: Time constraints (personal or institutional)?
   c. Probe: Others?
7. In your opinion, how much does the VA encourage professional development opportunities for VA providers? (Please explain.)
8. If you were to pursue a professional development opportunity, what would it look like for your VA supervisors, ambulatory care, and facility leadership support you in this?
   a. Probe: What type of support do feel is most needed? (financial, time, networking, mentorship, other)
9. If you were to pursue a professional development opportunity, how well would your PACT support you?
   a. Probe: What type of support would be most needed?
   b. Probe: To what extent would it be helpful for programs offering professional development opportunities to rural VA providers to engage your PACT in some way?
   c. Probe: What type of engagement would be most helpful?
10. What does “quality improvement” (or QI) mean to you in your practice?
11. Have you been involved in any projects to improve clinic workflow or processes with the goal of improving care to the patient?  If so, can you describe the project to me?
12. IF DID participate or lead QI:
a. How many disciplines/roles were represented on the team?
b. What was the outcome of your project?
c. Did you have access to mentorship or coaching?
d. What if any significant barriers did you experience in trying to complete the project?
e. How did you incorporate working on the QI project into your working schedule?

       IF DID NOT participate or lead QI:

a. What barriers to leading or participating in quality improvement projects have you experienced?
b. What would increase your interest in leading or participating in a quality improvement project?

13. (If you have participated in QI) Please describe what you learned through your participation in a QI or similar initiative.
    a. Probe: What if any new skills did you develop?
    b. Probe: What in your view is the value of a QI initiative?
    c. Probe: Did you feel that your confidence increased after participating in a QI project?  (Please explain.)

14. [Refer to their response to the survey question number 8] How do you see QI skill development as a professional development opportunity, or not?
15. How would a professional development opportunity focused on quality improvement leadership, skills, tools, and knowledge add value to your practice?
16. What would make QI initiatives more relevant to you and other VA providers?

Supplement D: FOCUS GROUP GUIDE FOR AMBULATORY CARE LEADERS

Introduction, Brief welcome, purpose of the study, confidentiality statement, verbal consent.

Main Questions:

1. Understanding VA ambulatory care leadership perspectives related to professional development in general, and in quality improvement initiatives, for rural VA providers?
2. What types of professional development opportunities are offered to the providers you supervise through the VA during your time working at your rural CBOCs?
   a. E.g., clinical skills development, professional skills development, quality improvement, leadership.
3. Which types have been most popular among rural providers?
4. What barriers have you noticed may impede rural providers from taking advantage of opportunities?
5. What incentives are there for providers to take advantage of professional development opportunities?
   a. Probe: Any disincentives?
6. What professional development opportunities do you feel are most valuable or relevant to rural VA providers?
7. Do you feel that you are as aware as you could be about professional development opportunities for VA primary care providers?
   a. Probe: What would be the best forum for communicating these opportunities with leadership?
8. As ambulatory care leadership, how equipped did you feel to be able to lead, mentor, or coach rural VA providers in leadership and quality improvement?
   a. Probe: What skills did you develop to enable you to fulfill this role?
   b. Probe: What additional skills would you find useful to you to be able to mentor, or coach primary care providers in their professional development?
9. What resources and processes are in place within your VA to provide coaching or mentorship to primary care providers?
   a. Probe: How are these resources made accessible to rural VA providers?
10. How are professional networks for VA primary care providers encouraged?
    a. Probe: How are these processes similar or different for rural VA providers?
11. What possibilities exist for career advancement for rural VA providers?
    a. Probe: Are there opportunities within the VA for providers with unique skill sets to advance?
12. One common barrier raised by providers is dedicated time for participation in QI professional development, QI project design and completion.
13. What would dedicated time for professional development look like from your standpoint in leadership?
14. What types of resources are available to providers who are interested in quality improvement; is it different for providers in rural CBOCs?
15. How do providers who are interested in QI efforts at the local clinic level access the data they need to measure improvement?
16. In what ways could professional development play a role in recruitment to rural VA CBOCs?
17. In what ways could professional development play a role in retention in rural VA CBOCs?

Supplement E – INTERVIEW GUIDE FOR FACILITY LEADERSHIP

Introduction

Brief welcome, purpose of study, confidentiality statement, verbal consent

Main Questions

1. What types of professional development opportunities in quality improvement or leadership are offered through the VA to providers?
2. Are these opportunities also available for ambulatory care providers in rural CBOCs?
   a. Probe:  Which opportunities were most often pursued by these providers?  (Which type were most popular among providers?)
3. What do you see as the most prominent barriers to VA providers taking advantage of professional development opportunities?
   a. Probe: How are these similar or different for rural providers?
4. What VA professional development opportunities do you feel are most valuable or relevant to rural VA providers?
5. What would be the best forum for communicating opportunities with you?
6. How equipped do you feel ambulatory care leaders are to be able to mentor, or coach rural VA providers in leadership and quality improvement?
7. What resources and processes are in place within your VA to provide coaching or mentorship to primary care providers?
   a. Probe: How are these resources made accessible to rural VA providers?
8. How are professional networks for VA providers encouraged?
   a. Probe: How are these processes similar or different for rural VA providers?
9. What possibilities exist for career advancement for rural VA providers?
   a. Probe: Are there opportunities within the VA for providers with unique skill sets to advance?
