# Supplementary material for: “Kind of Feel Like the Lone Outpost”: A Qualitative Exploration of Barriers to Professional Development for Rural Primary Care Providers at the Veterans Health Administration
Source: J Rural Health. 2026 Aug 18;42(3):e70206. doi: 10.1111/jrh.70206 (PMC13483286; doi:10.1111/jrh.70206)
Supplement: Supplementary file 3 [file JRH-42-0-s003.docx]

**Supplement F. Illustrative Quotations by Theme**

*(****Companion to Manuscript Results****)*

*Participant role descriptors include clinical role, gender, credentials, and region (rural Veterans Health Administration region where the participant practiced). Regions are omitted for ambulatory care leaders and facility leaders to protect confidentiality. PCP = primary care provider; MD = medical doctor; NP = nurse practitioner; PA = physician assistant.*

| **Theme / Subtheme** | **Illustrative Quote** | **Participant Role** |
| --- | --- | --- |
| **1. Ineffective or delayed communication of PD** | *“I don’t even know where I would get information (PD).”* | PCP (Female MD, Northeast) |
|  | *“I would feel completely frustrated when I would receive communication about a webinar that was happening in a week, and my schedule is already completely booked.”* | PCP (Female MD, Midwest) |
|  | *“Show consideration—if you are sending the opportunity the week before, that may be okay for someone with a lot of administrative time, but if I’m in clinic I have to give 45 days’ advanced notice. It seems inconsiderate, it comes off that way.”* | Ambulatory Care Supervisor (Male MD) |
| **2a. Rural clinic capacity constraints: Understaffing and inadequate patient coverage** | *“Unfortunately for CBOCs, they are always short-staffed. We do have a float provider here, but she’s hardly ever here because she’s floating around at other places.”* | PCP (Female PA, Midwest) |
|  | *“[I need] more coverage of my [patient] panel to do any sort of training [PD].”* | PCP (Female MD, Northeast) |
|  | *“If [the] VHA really believes in this model of care [Patient-Aligned Care Teams], it needs to take intentional steps as an organization to fully staff these teams.”* | Facility Leader (Male MD) |
| **2b. Rural clinic capacity constraints: Insufficient protected time for PD** | *“I think the main consideration is always the time factor, and how it’s going to impact your panel management and your colleagues.”* | PCP (Male MD, South) |
|  | *“I cannot even cancel a clinic to attend an in-service. Way too much need. It’s really hard to imagine even taking annual leave.”* | PCP (Female MD, Northeast) |
|  | *“If it requires significant time—more than an hour or two—they’re trying to balance what they will gain from the training with their clinical load.”* | Ambulatory Care Supervisor (Female MD) |
|  | *“If you have one nurse for three teams, it [PD] is very challenging to do.”* | Ambulatory Care Supervisor (Male MD) |
| **3a. Higher opportunity costs: High rural care coordination workload** | *“It is very, very challenging to do well, to be a good [rural] primary provider. Care coordination is really challenging…it’s really hard to read records from [non-VHA] institutions that you’re not part of and try to figure out what’s going on.”* | PCP (Female MD, Northeast) |
|  | *“The farther away your clinic is from your main VHA, the way higher likelihood that all of your specialists will be through CITC (community care). It’s a huge workload that is not being studied… and it just sucks up so much time.”* | Ambulatory Care Supervisor (Female MD) |
| **3b. Higher opportunity costs: Conflicting metrics** | *“I was retrained on joint injections, knee injections, shoulder injections, and battlefield acupuncture, which was great, but I got so busy doing those things, that my access [metric] became poor… it’s a great service, but there’s a payoff.”* | PCP (Female MD, Midwest) |
|  | *“If you are going to value it [PD], you have to demonstrate that value by offering the time [to do PD] and not being as focused on the numbers [patient quotas].”* | Ambulatory Care Supervisor (Male MD) |
| **3c. Higher opportunity costs: Burnout** | *“I’m not in a position where I think I could do an additional ‘something.’”* | PCP (Female MD, Northeast) |
|  | *“It’s really hard when you have a fully paneled provider who is somewhat burnt-out just seeing patients, it’s really hard for them to be able to take advantage of the opportunities that the VHA does have to offer.”* | Ambulatory Care Supervisor (Female MD) |
|  | *“One of my goals is to not leave primary care because of burnout… I was given a lot of advice, through [non-VHA] networks, that I should negotiate clinic part-time if I want to stay in primary care for the next 30-40 years.”* | PCP (Female MD, Northeast) |
|  | *“When you’re crunched for time and have all of these other things going on, it’s hard to think in the long term about career development.”* | PCP (Male MD, West) |
| **4. Limited geographical access to PD** | *“The problem is you just can’t get there when you work in a rural area.”* | PCP (Female NP, West) |
|  | *“If I have to go to main VHA, it’s a 2 hour drive each way.”* | PCP (Female MD, South) |
|  | *“If it has to be in person, which is really great, having more diverse geographic opportunities would be helpful.”* | Ambulatory Care Leader (Male MD) |
| **5a. Professional isolation: Lack of professional networks** | *“You feel pretty isolated [working in a rural CBOC], so opportunities to network would be something I am looking for.”* | PCP (Female NP, West) |
|  | *“[Rural PCPs] kind of feel like the lone outpost.”* | Ambulatory Care Supervisor (Male MD) |
| **5b. Professional isolation: Disconnect between rural clinics and main campus** | *“[There is a] disconnect between the ‘boots on the ground’ people, and those doing research, QI, [and] administration… intentionality is needed to connect people in power, or research, to those people working in rural clinics.”* | PCP (Female MD, Northeast) |
|  | *“We have an academic affiliate, and providers there have much more protected time, do less clinic, and have better opportunities for professional advancement.”* | Ambulatory Care Supervisor (Male MD) |
| **5c. Professional isolation: Limited developmental relationships** | *“Mentorship is always good, [but] I don’t know anybody who really gets mentored as a PCP.”* | PCP (Female MD, Northeast) |
|  | *“Not having someone who knows the ropes [is challenging]. [Someone] who can tell you, ‘Here are some directions you could go.’ No one mentors you out here. I wouldn’t know where to start.”* | PCP (Female NP, West) |
|  | *“[There is not enough] admin time to support the CBOCs properly.”* | Ambulatory Care Supervisor (Female MD) |
|  | *“If you are 100% clinical, how are you supposed to be a mentor for somebody? It’s something we would like to do, but we just haven’t had the resources.”* | Ambulatory Care Supervisor (Male MD) |
|  | *“There’s no mentorship, [but] that would be awesome if someone sat with you and went over your career goals.”* | PCP (Female MD, Midwest) |
| **5d. Professional isolation: Limited pathways for rural career advancement** | *“Advancement is hard because it typically requires a move, other than designation as a medical director [of CBOC].”* | PCP (Male MD, West) |
|  | *“As physicians when we hear the word ‘leadership,’ we think we have to be a service chief or a C-suite person and get away from patient care. I think what we need is BOTH AVENUES—management and team/initiative leadership.”* | Facility Leader (Male MD, South) |
| **6. Perceived devaluation of PCP role** | *“I understand that this is a bureaucratic system and that there is a hierarchy, and it is important to follow the rules. But I also think there is definitely room for improvement to recruit people to more than ‘just a job.’”* | PCP (Male MD, South) |
|  | *“As much as I love my job, I feel like a factory worker, and my other docs have said that.”* | PCP (Female MD, South) |
|  | *“People want professional satisfaction more than they want money.”* | Facility Leader (Male MD) |
|  | *“I’ve just been a ‘worker bee’ in primary care for the last twenty-some years.”* | PCP (Female MD, Midwest) |
